# A molecular toolbox for fast and convenient diagnosis of emerging and reemerging bacterial pathogens causing fever of intermediate duration

**DOI:** 10.1007/s10096-024-04760-8

**Published:** 2024-01-25

**Authors:** Elva Vega-García, Génesis Palacios, José A. Pérez, Mónica Vélez-Tobarias, Ana María Torres-Vega, Carlos Ascaso-Terrén, Emma Carmelo

**Affiliations:** 1 Instituto Universitario de Enfermedades Tropicales y Salud Pública de Canarias (IUESTPC), Universidad de La Laguna (ULL), Avenida Astrofísico Francisco Sánchez S/N, 38200 La Laguna, Tenerife Spain; 2 Departamento de Bioquímica, Microbiología, Biología Celular y Genética, Facultad de Ciencias, Universidad de La Laguna, La Laguna, Tenerife Spain; 3 Servicio de Medicina Interna, Hospital Universitario de La Palma (HULP), Breña Alta, La Palma Spain; 4 Servicio de Medicina Interna, Hospital Insular Ntra. Sra. de los Reyes (HINSR), Valverde, El Hierro Spain; 5 Departamento de Fonaments Clínics, Universitat de Barcelona, Barcelona, Spain; 6 Departamento de Obstetricia y Ginecología, Pediatría, Medicina Preventiva y Salud Pública, Toxicología, Medicina Legal y Forense y Parasitología, Universidad de La Laguna, La Laguna, Tenerife Spain

**Keywords:** Molecular toolbox, Fever of intermediate duration, Arthropod-borne bacteria, Real-time PCR, FRET probes, Molecular diagnostics

## Abstract

**Purpose:**

Fever of intermediate duration (FID) is defined as a fever in the community without a specific origin or focus, with a duration between 7 and 28 days. FID is often caused by pathogens associated with animal contact or their arthropods parasites, such as ticks, fleas, or lice. The purpose of this work is to design a collection of molecular tools to promptly and accurately detect common bacterial pathogens causing FID, including bacteria belonging to genera *Rickettsia*, *Bartonella*, *Anaplasma*, and *Ehrlichia*, as well as* Coxiella burnetii.*

**Methods:**

Reference DNA sequences from a collection of *Rickettsia*, *Bartonella*, *Anaplasma*, and *Ehrlichia* species were used to design genus-specific primers and FRET probes targeted to conserved genomic regions. For *C. burnetii*, primers previously described were used, in combination with a newly designed specific probe. Real-time PCR assays were optimized using reference bacterial genomic DNA in a background of human genomic DNA.

**Results:**

The four real-time PCR assays can detect as few as ten copies of target DNA from those five genera of FDI-causing bacteria in a background of 300 ng of human genomic DNA, mimicking the low microbial load generally found in patient’s blood.

**Conclusion:**

These assays constitute a fast and convenient “toolbox” that can be easily implemented in diagnostic laboratories to provide timely and accurate detection of bacterial pathogens that are typical etiological causes of febrile syndromes such as FID in humans.

## Introduction

Fever of intermediate duration (FID) is defined as a fever in the community without a specific origin or focus, with a duration between 7 and 28 days [1]. This febrile syndrome is frequently caused by pathogens associated with animal contact or their arthropods parasites. Zoonotic bacterial pathogens causing fever of intermediate duration (FID) are a significant public health concern due to their increasing incidence and geographic range, as well as their potential to cause severe illness and death [2]. In Spain, the most recent studies include Q fever and rickettsiosis as the most reported bacterial causes of FID [3–5]; similarly, other viral pathogens, such as cytomegalovirus (CMV) or Epstein-Barr virus (EBV), and more recently SARS-CoV-2, have been described as causative agents of fever and particularly FID all over the world [3–6]. Particularly, there is a group of bacterial pathogens including *Rickettsia* spp., *Coxiella burnetii*, *Bartonella* spp., and species of *Anaplasma/Ehrlichia* that are typical etiological causes of febrile syndromes associated to contact with animals and their arthropod parasites (fleas, ticks, etc.). These pathogens can affect both human and animal health, and many of them have animal vectors and/or reservoirs.

Species of *Rickettsia* genus are etiological agents of rickettsiosis in humans and animals and are traditionally divided into the spotted fever group (SFG) and the typhus group (TG). In Europe, *Rickettsia* species transmitted by *Ixodidae* ticks include *Rickettsia conorii*, *Rickettsia helvetica*, *Rickettsia monacensis*, *Rickettsia massiliae*, and *Rickettsia aeschlimannii*, among others [7]. Infection by these species of the SFG in humans causes from mild (such as fever, rash, and eschar) to severe and life-threatening clinical signs, depending on the species [8]. Flea-borne species comprise *Rickettsia typhi* (causing murine typhus) and *Rickettsia felis*, widely found in fleas but rarely described as a human pathogen [7].

Several species of the *Anaplasmataceae* family are tick-borne gram-negative obligate intracellular bacteria that have been found causing acute infection in humans and a broad range of wild and domestic mammals, including horses, dogs, cats, deer, goats, sheep, and cattle [9, 10]. *Anaplasma phagocytophilum* (causing Human Granulocytic Anaplasmosis), *Ehrlichia chaffeensis* (causing Human Monocytic Ehrlichiosis), and *Ehrlichia ewingii* (agent of Human Granulocytic Ehrlichiosis) are the main species of this family reported as human pathogens, but other species (*Anaplasma marginale*, *Anaplasma centrale*, *Anaplasma bovis*, *Anaplasma ovis*, *Anaplasma platys* or *Ehrlichia muris*) are typical animal pathogens and should not be discarded as etiological agents of disease [11].

Q fever is an emerging zoonotic disease caused by the intracellular bacterium *Coxiella burnetii*, capable of infecting humans and a wide spectrum of animals worldwide, including wild and domestic mammals, birds, reptiles, and arthropods [12, 13]. Infection generally occurs by inhalation of contaminated aerosols during contact with animals, particularly their birth products, milk, faeces, and urine, although ticks also play a role in the wild and peridomestic epidemiological cycles of *C. burnetii* [14]. In humans, Q fever presentation can range from mildly symptomatic (a self-limited febrile illness) to a fatal disease [12].

*Bartonella* spp. comprise at least 35 potentially zoonotic species of Gram-negative bacilli included in the class *Alphaproteobacteria* [15]. The most frequently reported human or animal pathogen of this genus is *Bartonella henselae*, the causative agent of Cat Scratch Disease [16]. Infections cause lymphadenopathy, and sometimes endocarditis, febrile illness, hepatosplenic abscesses, and bacillary angiomatosis, among others [16, 17]. *Bartonella* spp. are mainly transmitted by contact with fleas and lice faeces, although several animals have been described as hosts and reservoirs of species of this genus [15]. *Bartonella* infections are considered emerging and re-emerging infections in humans.

Serological testing is the most common method for the diagnosis of patients suffering FID, although the interpretation of results is hindered by the need for paired specimens, low or delayed serological responses in some patients, and cross-reactivity among closely related bacterial species [1, 18]. Pathogen DNA detection using conventional and quantitative real-time PCR analysis is helpful if performed on blood or tissue samples in the first 5–10 days after symptom onset [8, 19–21], but there is still a need for improved laboratory capacity, diagnostic tools, and awareness among clinicians to detect and control vector-borne diseases from a “one health” perspective [22].

In this paper, a collection of four real-time PCR assays is designed to detect as few as ten copies of target DNA of *Rickettsia* spp., *Bartonella* spp., *Anaplasma/Ehrlichia* spp., and *Coxiella burnetti* DNA in a background of human genomic DNA, as a proxy for the diagnostic procedure on human samples. All assays follow the same PCR thermal profile and are coupled to an end-point melting analysis of probe/amplicon duplexes, in order to provide additional information about the detection process and, in some cases, the pathogen involved. These assays constitute a fast and convenient “toolbox” that can be easily implemented in diagnostic laboratories to provide timely and specific detection of bacterial pathogens that are typical etiological causes of febrile syndromes such as FID in humans.

## Methods

### Genomic DNA samples

The design of this work conforms to European legal and ethical standards and was approved by Comité de Etica de la Investigación con Medicamentos (CEIm) of Hospital Universitario de Canarias (Tenerife, Spain) and coded 2017_81. All patient’s written consents were obtained.

Human blood samples were collected by venous puncture in vials containing EDTA as anticoagulant. Genomic DNA (gDNA) was purified from 250-µL aliquots of whole blood using the E.Z.N.A.® Blood DNA Mini Kit (Omega Bio-Tek), following manufacturer’s instructions and eluting gDNA with 60 µL of 10 mM Tris–HCl, pH 8.0. The concentration and purity level of these gDNA preparations were estimated by spectrophotometry, and then concentration of gDNA samples was adjusted to 50 ng/µL using the same buffer.

Human gDNA used as negative control or as nucleic acid background was purified from a healthy volunteer. The gDNA samples used as positive controls for *Anaplasma phagocytophilum* and *Ehrlichia chaffeensis* were obtained from a mixture of 150 µL of whole blood from a healthy volunteer and 100 µL of a commercial bacterial suspension (Exact Diagnostics), containing approximately 1000 cells of the corresponding species. Positive controls for *Coxiella burnetii* and *Rickettsia typhi* were gDNA samples purified from infected patients in acute phase, which were subsequently diagnosed through immunological testing (COXIELLA BURNETII I + II IFA IgG/IgM/IgA and RICKETTSIA TYPHI IFA IgG, Vircell Microbiologists). *Rickettsia conorii* and *Bartonella henselae* controls consisted of gDNA purified from a culture of Vero cells infected with *R. conorii* or from blood agar culture of *B. henselae* (AmpliRun®—Vircell), which were afterwards mixed with the human gDNA negative control, in such a way that 6 µL of this mix contained approximately 1000 bacterial genome equivalents in a background of 300 ng human gDNA.

### Design of amplification primers and probes

Different genomic loci and a set of reference DNA sequences were considered for designing detection assays based on real-time PCR (Table 1). For the monospecific genus *Coxiella*, we used the primers described previously by Willems et al*.* [23], which are targeted to a repeated DNA element of the *C. burnetii* genome. The genomic targets for *Rickettsia* and *Bartonella* genera were the internal transcribed spacer (ITS) between genes encoding 23S-5S or 16S-23S ribosomal RNAs (rRNA), respectively. In the case of *Bartonella*, we modified the primers designed by Parra et al*.* [24]. Oligonucleotides for *Anaplasma* and *Ehrlichia* detection were designed on the gene encoding 16S rRNA (*rrs* gene).

**Table 1:** Genomic loci and species considered for designing DNA oligonucleotides

Loci	Species
Transposon-like sequence IS1111	*Coxiella burnetii*
23S-5S rRNA ITS	*Rickettsia aeschlimannii*; *R. africae*; *R. akari; R. amblyommatis*; *R. australis*; *R. bellii*; *R. conorii*; *R. felis*; *R. heilongjiangensis*; *R. helvetica*; *R. honei*; *R. japonica*; *R. massiliae*; *R. monacensis*; *R. mongolotimonae*; *R. montanensis*; *R. parkeri*; *R. prowazekii*; *R. raoultii*; *R. rickettsii*; *R. slovaca*;* R. typhi*
16S-23S rRNA ITS	*Bartonella alsatica*; *B. ancashensis*; *B. australis*; *B. bacilliformis*; *B. doshiae*; *B. elizabethae*; *B. grahamii*; *B. henselae*; *B. kosoyi*; *B. krasnovii*; *B. quintana*; *B. schoenbuchensis*; *B. taylorii*; *B. tribocorum*;* B. vinsonii*
16S rRNA	*Anaplasma bovis*; *A. marginale*; *A. phagocytophilum*; *A. platys*; *Ehrlichia canis*; *E. chaffeensis*; *E. ewingii*; *E. muris*;* E. ruminantium*

First, reference sequences from *R. conorii*, *B. henselae*, *A. phagocytophilum*, and *E. chaffeensis* were used to identify homologous sequences within the corresponding genus by BLASTn searching. Chosen sequences were downloaded from GenBank database (NCBI) and aligned with MEGA X software [25]. Conserved regions in the alignment of DNA sequences were selected for the design of oligonucleotides used as primers or hybridization probes with the software Gene Runner version 6.5.52 (Hastings Software Inc.). The melting temperature (*Tm*) of the oligonucleotides was calculated as the average of predictions made by three different applications: Gene Runner, Oligo Calc (http://biotools.nubic.northwestern.edu/OligoCalc.html), and OligoAnalyzer™ Tool (https://eu.idtdna.com/pages/tools/oligoanalyzer). OligoAnalyzer™ Tool was also used to estimate the decrease in oligonucleotide *Tm* caused by different mismatches.

### Real-time PCR

Real-time PCR assays were performed using the thermal cycler LightCycler® 480 (Roche). Each 50-µL reaction contained 300 ng of gDNA template, 1X reaction buffer (Thermo Fisher), additional MgCl_2_ (depending on amplicon), 0.2 mM of dNTP mix, 0.2 µM of forward primer, 0.2 µM of reverse primer, 0.2 µM of each probe, and 0.5 µL of Phire Hot Start II DNA Polymerase (Thermo Fisher). It is highly recommended to use a thermostable DNA polymerase lacking 5′-to-3′ exonuclease activity in order to avoid probe degradation.

PCR thermal profile consisted of an initial denaturation at 98 °C for 30 s, followed by 45 amplification cycles with denaturation at 98 °C for 5 s, annealing at 56 °C for 5 s, and extension at 72 °C for 10 s. Fluorescence emission was measured during the annealing step using excitation/emission filters with a wavelength of 483/670 nm. After amplification, probe melting analysis was performed with the following thermal profile: 95 °C for 1 min, 40 °C for 90 s (55 °C for 10 s in *Anaplasma*/*Ehrlichia* assay) and a continuous fluorescence monitoring from 40/55 to 95 °C with three acquisitions/°C.

### Construction of standard curves

First, the specificity of positive PCRs from gDNA controls was confirmed by amplicon sequencing. Selected amplicons were purified with MicroElute® Cycle-Pure Kit (OMEGA Bio-Tek) following manufacturer’s instructions and quantified by spectrophotometry. Next, purified amplicons were subjected to serial dilution from 10^6^ to 10^1^ molecules per 6 µL, using human gDNA control at 50 ng/µL as diluent. Standards of amplicon concentration were assayed in triplicate by real-time PCR. Fluorescence thresholds used for estimating quantification cycles (Cq) [26] were manually set. The logarithm of the number of amplicon molecules for each standard, and the corresponding average *Cq* were entered into an Excel spreadsheet for calculating correlation coefficients (*R*^2^). Amplification efficiencies for each primer pair were calculated from standard curves as *E* = 10^(−1/slope)^ − 1 [26].

## Results

The set of oligonucleotides selected for the detection of these five genera of arthropod-related bacterial pathogens by real-time PCR, and subsequent identification of the corresponding species by amplicon sequencing, is shown in Table 2. Although highly conserved genomic loci were used to design oligonucleotides for *Rickettsia*, *Bartonella*, and *Anaplasma*/*Ehrlichia* detection assays, it was necessary to incorporate degenerate positions in the sequence of some primers to deal with the high number of different species within these bacterial genera (Table 1). In our real-time PCR assays intended for *Coxiella*, *Rickettsia*, and *Bartonella* detection, Fluorescence Resonance Energy Transfer (FRET) occurs between a labelled primer and a probe, whereas in the *Anaplasma*/*Ehrlichia* assay FRET occurs between two labelled probes [27, 28].

**Table 2:** DNA oligonucleotides toolbox for detection and characterization of different bacteria causing fever of intermediate duration

Bacterial genus		Oligonucleotides							Amplicon length (bp)
	Use^b^	Binding Region	Positions (5’-to-3’)^c^	Sequence^d, e^	Length (nt)	Modification		Tm (ºC)^f^	
*Coxiella*^a^	A	Transposase gene (IS1111)	200-221	TATGTATCCACCGTAGCCAG**T**C	22	FAM		64.0	686
	A		885-865	CCCAACAACACCTCCTTATTC	21			63.2	
	P		240-223	ACCACGCAGCCCACCTT**A**	18	Cy5		65.6	
	S		240-256	TGATGGAAGCGTGTGGA	17			58.3	
*Rickettsia*	A	23S rRNA gene	32-56	TGTAGCTAACYGATACTAATAGC**T**C	25	FAM		58.8-63.0	302-372
	A	5S rRNA gene	379-359	GATCGTGTGTTTCACTCATGC	21			62.2	
	P	23S rRNA gene/ intergenic spacer	81-57	AATCTCACAGCAAAGTAAATCAAT**C**	25	Cy5		64.7	
	S	5S rRNA gene/ intergenic spacer	365-346	CTCATGCTATRACCACCAAG	20			57.2-59.7	
*Bartonella*	A	23S rRNA gene	1,434,678-1,434,696	GSATCCACCAAATGCCC**T**T	19	FAM		61.8-62.5	245-559
	A	intergenic spacer	1,435,059-1,435,035	GTGAAGAGAAGATATATTCARACAT	25			57.2-58.7	
	P	23S rRNA gene/ intergenic spacer	1,434,744-1,434,721	GGCAATGAGAACGATCAAGTGTC**T**	24	Cy5		65.2	
	S		1,434,690-1,434,712	TGCCCTTAAGACACTTGATCGTT	23			62.8	
*Anaplasma/Ehrlichia*	A	16S rRNA gene	89-118/ 131-160	GRATAGCCAYTAGAAATGATGGGTAATACT	30			64.4-66.6	572
	A		660-637/ 702-679	AGTGTCAGTATCGRACCAGAYAGC	24			63.0-67.0	
	P		220-243/ 262-285	GGTCTGAGAGGACGATCAGCCAC**A**	24	Cy5		69.8	
	P		248-277/ 290-319	**A**ACTGAGATACGGTCCAGACTCCTACGGGA**A**	30	5’-FAM, 3’-P		72.8	
	S		634-615/ 663-644	CCTTCGCCACTGGTGTTCCT	20			63.8	

^a^Amplification primers described in Willems et. al [23]

^b^A, amplification primer; S, sequencing primer; P, probe

^c^Nucleotide positions relative to reference sequences from GenBank of *C. burnetii* (M80806), *R. conorii* (AY125012), *B. henselae* (CP020742), *A. phagocytophilum* (AF470701) and *E. chaffeensis* (U60476)

^d^Nucleotides with an attached fluorescent dye (Cy5/FAM) or blocked with phosphate (P) are highlighted in bold

^e^Degenerate positions: R (A or G); S (G or C); Y (C or T)

^f^Theoretical melting temperature (Tm) assuming 100% complementarity between the oligonucleotide and its target. Minimum and maximum Tm values are indicated for degenerate primers

First, each detection assay was tested with its corresponding positive control; 300 ng of human gDNA was included in each amplification reaction, in order to overcome the low microbial load generally found in patient’s blood and therefore increase the likelihood of successful detection of patients with rickettsioses. Since DNA acts as a sequestering agent of Mg^2+^, it was necessary to optimize the concentration of MgCl_2_ in the amplification reaction. In this sense, the lowest *Cq* values were obtained with 4.5 mM MgCl_2_ for *Coxiella* and *Anaplasma*/*Ehrlichia* PCR assays, 6 mM for *Rickettsia* and 6.5 mM for *Bartonella* assays. The sequences of the amplicons obtained in these PCRs matched with the correct genomic loci and the corresponding bacterial species.

**Fig. 1:**
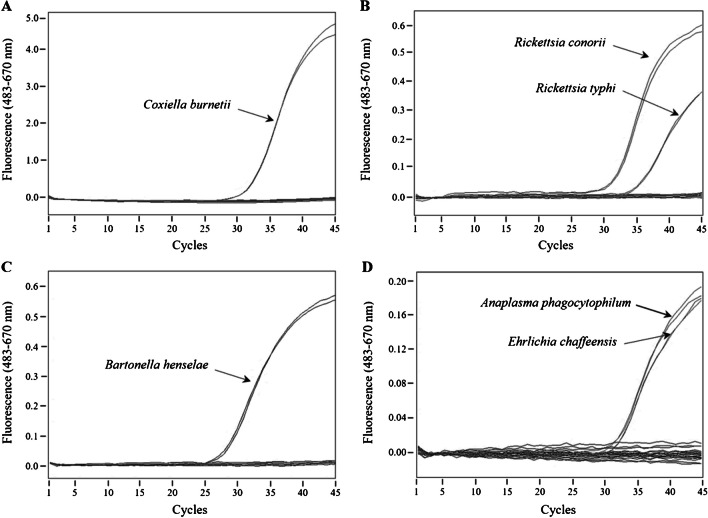
Specificity assessment of the real-time PCR assays. Detection tests for *Coxiella* (**A**), *Rickettsia* (**B**), *Bartonella* (**C**), and *Anaplasma*/*Ehrlichia* (**D**) were run in duplicate with gDNA samples from the following bacterial species: *C. burnetii*, *R. conorii*, *R. typhi*, *B. henselae*, *A. phagocytophilum*, and *E. chaffeensis*. Two replicates using human gDNA as template and two negative controls (water) were also included

**Fig. 2:**
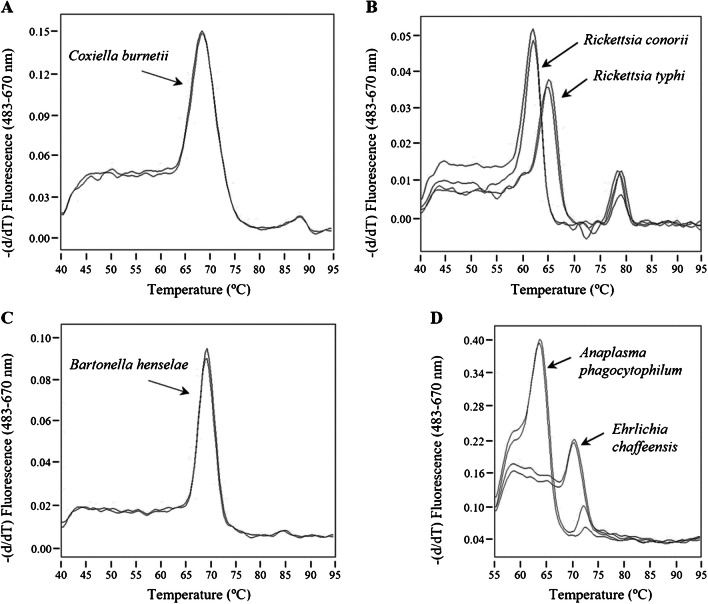
Melting curves of probe/amplicon duplexes. End-point analysis was performed on positive PCRs shown in Fig. 1. The annotated *Tm* values were 69.4 °C (*C. burnetii*), 62.0 °C (*R. conorii*), 65.2 °C (*R. typhi*), 69.2 °C (*B. henselae*), 63.7 °C (*A. phagocytophilum*), and 71.5 °C (*E. chaffeensis*)

To check the specificity of the real-time PCR assays in terms of taxon detection, each one was confronted to the complete panel of gDNA samples described in Methods section, including gDNA from a healthy human donor. As can be seen in Fig. 1, amplification signal was only observed with the gDNA sample from the bacterial genus for which the PCR test was devised. Furthermore, end-point melting analysis of probe/amplicon duplexes provided more confidence to the detection assay and, in some cases, additional information about the pathogen (Fig. 2). While for the 15 *Bartonella* species listed in Table 1 the same *Tm* value is expected, a mismatch in the mentioned DNA duplex decreases the *Tm* registered for *R. conorii* (and the expected for *R. akari* and *R. australis*) in comparison to *R. typhi* (Fig. 2) and the 18 remaining *Rickettsia* species in Table 1. Also due to a single mismatch, the *Tm* observed with *Anaplasma* spp. is lower than with *Ehrlichia* spp., so that both genera could be detected and differentiated in a single assay (Fig. 2).

The technical sensitivity of the detection assays and their suitability for quantification of the bacterial load were assessed with a series of concentration standards from 10^1^ to 10^6^ copies of target DNA in a sample of 6 µL. All four tests could detect as low as ten copies of the corresponding DNA sequence, with *Cq* values between 33.4 and 35.7 depending on the assay. In general, the calibration curves showed a very good linearity (*R*^2^ > 0.99) over the whole range of tested concentrations, but some slight inaccuracy may arise when it is required to quantify less than 100 copies of the target sequence (Table 3). The amplification efficiencies of the different real-time PCR assays, calculated from the calibration curves, were maximal (1 or 100%) or near maximal (Table 3).

**Table 3:** Dynamic range for quantification and amplification efficiency of the real-time PCR assays

Target	Correlation coefficient (*R*^2^)		Amplification efficiency
	10^1^–10^6^ range ^a^	10^2^–10^6^ range	
*Coxiella burnetii*	0.9999	0.9999	0.990
*Rickettsia typhi*	0.9993	0.9996	1
*Bartonella henselae*	0.9946	0.9973	1
*Anaplasma phagocytophilum*	0.9969	0.9993	1
*Ehrlichia chaffeensis*	0.9968	0.9994	0.987

^a^Target copies in 6 µl of DNA sample

## Discussion

The set of primers and probes presented in this work potentially allows detection by real-time PCR of a wide range of bacterial species belonging to five genera, typical causative agents of FID. This collection enables diagnostic laboratories with molecular capacities to provide detection of *Rickettsia* spp., *Bartonella* spp., *Anaplasma/Ehrlichia* spp., and *C. burnetti* DNA in human blood samples in just a few hours, providing timely diagnosis during the acute phase of the disease. Moreover, since all assays share the same PCR thermal profile, all can be performed simultaneously, using 56 °C as the adequate annealing temperature during PCR, therefore improving turnaround time and patients’ management.

The use of specific fluorescent primers and probes for each genus is a guarantee that the genuine DNA sequence is being amplified in all these real-time PCR assays, and the target sequence is present in the DNA sample used as template. Furthermore, FRET probes used in this work provide an additional verification for amplification specificity, ruling out spurious or artifactual generation of fluorescence through the melting analysis of probe/amplicon duplexes. It should be noted that *Tm* values predicted in silico (Table 2) are algorithm-dependent and they can differ appreciably from those calculated empirically. Since actual *Tm* values are affected by the particular composition of the PCR mix, they should be annotated after running amplification reactions with validated positive controls as template in any laboratory that uses this type of DNA probes.

After a positive result using the real-time PCR assays presented in this work for the detection of *Rickettsia*, *Bartonella*, and *Anaplasma*/*Ehrlichia*, particular species can be identified by amplicon sequencing, since all bacteria species mentioned in Table 1 can be clearly differentiated in this way. Perhaps the unique challenging case would be to distinguish between *A. phagocytophilum* and *A. platys*, because only three nucleotide substitutions are expected in the 572-bp amplicon derived from the coding sequence of *rrs* gene.

A widely used procedure to address the technical sensitivity (detection limit) of a real-time PCR assay is using a standard curve made up with cloned target DNA sequences (i.e., amplicons or plasmids). The correlation between the absolute quantification using standard curves and actual bacterial load depends on the target: since the corresponding targets are single copy sequences in the genome of *Rickettsia*, *Anaplasma*, and *Ehrlichia* species, in these cases the number of target copies in a sample equals the number of bacterial genome equivalents (1:1 ratio). However, for *Bartonella* spp., the ratio is 2:1 because the genome of this species has two copies of the 16S-23S rRNA ITS sequence. Finally, the copy number for the target transposon-like sequence oscillates between 3 and 103 in the genome of different *C. burnetii* strains, and about a third of the complete genome sequences of this species that have been deposited in databases (more than 170) have 20 copies of this mobile element. Although this introduces a substantial uncertainty in the quantification approach, at the same time confers a great technical sensitivity (potentially one genome equivalent) to the *C. burnetii* detection assay.

Several in-house DNA-based methods have been developed in the last decades for the detection of bacterial fever-causing pathogens in a variety of samples such as human blood or tissue biopsies, but also animal samples and arthropod vectors. These methods range from multiplex-conventional PCR combined to reverse-line blotting to nested PCR and amplicon sequencing [29–32]. The increasing availability of real-time PCR instruments in hospitals has turned real-time PCR into a very convenient point-of-care test for acute febrile patients [19]. In this context, the collection of primers and probes described in this paper constitute a useful “toolbox” that can be readily used in diagnostic laboratories to promptly and accurately detect common bacterial pathogens genera, including species responsible for causing febrile syndromes like FID in humans.

## Data Availability

Not applicable.

## References

[1] Guirao-Arrabal E, Muñoz-Medina L, Anguita-Santos F, Vinuesa-García D, Hernández-Quero J (2021). Empirical treatment with doxycycline of fever of intermediate duration. Eur J Clin Microbiol Infect Dis.

[2] Cantas L, Suer K (2014). Review: the important bacterial zoonoses in “one health” concept. Front Public Health.

[3] Espinosa N, Cañas E, Bernabeu-Wittel M, Martín A, Viciana P, Pachón J (2010). The changing etiology of fever of intermediate duration. Enferm Infecc Microbiol Clin.

[4] Parra Ruiz J, Peña Monje A, Tomás Jiménez C, Parejo Sánchez MI, Vinuesa García D, Muñoz Medina L (2008). Clinical spectrum of fever of intermediate duration in the south of Spain. Eur J Clin Microbiol Infect Dis.

[5] Huang C, Wang Y, Li X, Ren L, Zhao J, Hu Y (2020). Clinical features of patients infected with 2019 novel coronavirus in Wuhan. China Lancet.

[6] Liu BM, Martins TB, Peterson LK, Hill HR (2021). Clinical significance of measuring serum cytokine levels as inflammatory biomarkers in adult and pediatric COVID-19 cases: a review. Cytokine.

[7] Portillo A, Santibáñez S, García-Álvarez L, Palomar AM, Oteo JA (2015). Rickettsioses in Europe. Microbes Infect.

[8] Robinson MT, Satjanadumrong J, Hughes T, Stenos J, Blacksell SD (2019). Diagnosis of spotted fever group Rickettsia infections: the Asian perspective. Epidemiol Infect.

[9] Choubdar N, Karimian F, Koosha M, Nejati J, Oshaghi MA (2021). Hyalomma spp. ticks and associated Anaplasma spp. and Ehrlichia spp. on the Iran-Pakistan border. Parasit Vectors.

[10] Li H, Zheng Y-C, Ma L, Jia N, Jiang B-G, Jiang R-R (2015). Human infection with a novel tick-borne Anaplasma species in China: a surveillance study. Lancet Infect Dis.

[11] Karlsen A, Vojtek B, Mojžišová J, Prokeš M, Drážovská M (2020). Anaplasmosis in animals. Folia. Veterinaria.

[12] Angelakis E, Raoult D (2010). Q fever. Vet Microbiol.

[13] Celina SS, Cerný J (2022). Coxiella burnetii in ticks, livestock, pets and wildlife: a mini-review. Front Vet Sci.

[14] Eldin C, Mélenotte C, Mediannikov O, Ghigo E, Million M, Edouard S (2017). From Q fever to Coxiella burnetii infection: a paradigm change. Clin Microbiol Rev.

[15] Gandhi TN, Slater LN, Welch DF, Koehler JE (2015) Bartonella, including cat-scratch disease. In: Mandell, Douglas, and Bennett’s Principles and practice of infectious diseases. Elsevier, p. 2649–63

[16] Welch DF, Pickett DA, Slater LN, Steigerwalt AG, Brenner DJ (1992). Rochalimaea henselae sp. nov., a cause of septicemia, bacillary angiomatosis, and parenchymal bacillary peliosis. J Clin Microbiol.

[17] García JC, Núñez MJ, Castro B, Fernández JM, Portillo A, Oteo JA (2014). Hepatosplenic cat scratch disease in immunocompetent adults: report of 3 cases and review of the literature. Medicine (Baltimore).

[18] Brouqui P, Bacellar F, Baranton G, Birtles RJ, Bjoërsdorff A, Blanco JR (2004). Guidelines for the diagnosis of tick-borne bacterial diseases in Europe. Clin Microbiol Infect.

[19] Renvoisé A, Rolain J-M, Socolovschi C, Raoult D (2012). Widespread use of real-time PCR for rickettsial diagnosis. FEMS Immunol Med Microbiol.

[20] Bolaños-Rivero M, Carranza-Rodríguez C, Hernández-Cabrera M, Pisos-Álamo E, Jaén-Sánchez N, Pérez-Arellano J-L (2017). Utilidad del diagnóstico molecular precoz de fiebre Q y rickettsiosis en pacientes con fiebre de duración intermedia. Enferm Infecc Microbiol Clin.

[21] Bae M, Jin CE, Park JH, Kim MJ, Chong YP, Lee S-O (2019). Diagnostic usefulness of molecular detection of Coxiella burnetii from blood of patients with suspected acute Q fever. Medicine (Baltimore).

[22] Liu BM, Mulkey SB, Campos JM, DeBiasi RL (2023) Laboratory diagnosis of CNS infections in children due to emerging and re-emerging neurotropic viruses. Pediatr Res. 10.1038/s41390-023-02930-6 10.1038/s41390-023-02930-6 PMC12494120 38042947

[23] Willems H, Thiele D, Frölich-Ritter R, Krauss H (1994). Detection of Coxiella burnetii in cow’s milk using the polymerase chain reaction (PCR). Zentralbl Veterinarmed B.

[24] Parra E, Segura F, Tijero J, Pons I, Nogueras M-M (2017). Development of a real-time PCR for Bartonella spp. detection, a current emerging microorganism. Mol Cell Probes.

[25] Kumar S, Stecher G, Li M, Knyaz C, Tamura K (2018). MEGA X: Molecular evolutionary genetics analysis across computing platforms. Mol Biol Evol.

[26] Nolan T, Hands RE, Bustin SA (2006). Quantification of mRNA using real-time RT-PCR. Nat Protoc.

[27] Landt O (2001) Selection of hybridization probes for real-time quantification and genetic analysis. In: Meuer S, Wittwer C, Nakagawara K-I, editors. Rapid cycle real-time PCR. Berlin, Heidelberg: Springer Berlin Heidelberg, p. 35–41. 10.1007/978-3-642-59524-0_4

[28] von Ahsen N, Oellerich M, Schütz E (2000). Use of two reporter dyes without interference in a single-tube rapid-cycle PCR: alpha(1)-antitrypsin genotyping by multiplex real-time fluorescence PCR with the LightCycler. Clin Chem.

[29] Jado I, Escudero R, Gil H, Jiménez-Alonso MI, Sousa R, García-Pérez AL (2006). Molecular method for identification of Rickettsia species in clinical and environmental samples. J Clin Microbiol.

[30] Toledo A, Olmeda AS, Escudero R, Jado I, Valcárcel F, Casado-Nistal MA (2009). Tick-borne zoonotic bacteria in ticks collected from central Spain. Am J Trop Med Hyg.

[31] Pérez-Tanoira R, Ramos-Rincón JM, Martín-Martín I, Prieto-Pérez L, Tefasmariam A, Tiziano G (2020). Molecular Survey of Rickettsia spp., Anaplasma spp., Ehrlichia spp., Bartonella spp., and Borrelia spp. in Fleas and Lice in Ethiopia. Vector Borne Zoonotic Dis.

[32] Camprubí-Ferrer D, Oteo JA, Bottieau E, Genton B, Balerdi-Sarasola L, Portillo A et al (2023) Doxycycline responding illnesses in returning travellers with undifferentiated non-malaria fever: a European multicentre prospective cohort study. J Travel Med 18:30:taac094. 10.1093/jtm/taac094 10.1093/jtm/taac094 35932455

